# Associations between Acute and Chronic Effects of Exercise on Indicators of Metabolic Health: A Pilot Training Trial

**DOI:** 10.1371/journal.pone.0081181

**Published:** 2013-11-21

**Authors:** Anne Hecksteden, Teresa Grütters, Tim Meyer

**Affiliations:** Institute of Sports and Preventive Medicine, Saarland University, Saarbrücken, Germany; University of Bath, United Kingdom

## Abstract

**Background:**

The interindividual variability in the health-related efficacy of regular endurance training is high. This impedes on the individual optimization of preventive training prescriptions when optimal efficacy is intended. As a consequence, health-gains remain below what could be achieved. Practicable predictors of an individual’s responsiveness to physical exercise would offer a solution to this problem. Therefore, the present study aims to test the association of acute changes in indicators of metabolic health after an exhaustive exercise test and the respective chronic changes in response to an endurance training program.

**Methods:**

Fasting blood samples of healthy, untrained, non-smoking subjects (n=12, age 49 ± 7 years; body mass index 29 ± 4; maximum oxygen uptake 34 ± 7 ml·min^-1^·kg^-1^) were collected before and 1 hour after an exhaustive exercise test as well as after a 4 week supervised training period (walking / running 4 times per week at 60 % heart rate reserve).

**Results:**

A close linear relationship between acute and chronic changes could be demonstrated for insulin concentration (p=0.001; r=0.83), the fasting indicator of insulin sensitivity HOMA-IR (p<0.001; r=0.78) and non-esterified fatty acid concentration (p=0.001; r=0.88). No association became apparent for standard blood lipid parameters.

**Conclusion:**

It is concluded that the magnitude of acute exercise-induced changes in indicators of insulin sensitivity and non-esterified fatty acid concentration is a promising candidate for the prediction of chronic training induced changes in the respective parameter. However, further studies are needed to assess predictive accuracy.

**Trial Registration:**

www.clinicaltrials.gov NCT00934206 http://www.clinicaltrials.gov/ct2/show/NCT00934206

## Introduction

The effectiveness of exercise for disease prevention is beyond dispute as numerous epidemiological and interventional studies have documented the association of physical activity with lower morbidity and mortality and reductions in cardiovascular risk factors [1–3]. However, even within homogenous groups the magnitude of training effects (on performance as well as health-related parameters) varies widely and includes low- and even non-responders [4]. This impedes on the individual optimization of training prescriptions if optimal efficacy is intended. Practicable predictors of an individual’s responsiveness to regular physical exercise may help to solve this problem. From the clinical perspective this would offer the opportunity to prescribe targeted doses of exercise and improve coordination of exercise and pharmacological therapies. Moreover, from the scientific perspective, such predictors may be used as stratification criteria in order to improve the internal validity of training studies. 

In search of predictors for individual, health-related training efficacy it is noticeable that for several parameters acute changes after a single bout of exercise are similar to chronic changes after a corresponding training program. Important examples are decreased blood pressure [1,5] and increased insulin sensitivity [6]. An association between the two phenomena would make acute changes after a single, standardized bout of exercise a promising predictor of an individual’s responsiveness to physical exercise. For blood pressure such an association has been demonstrated recently by two independent training trials [7,8]. However, to the best of our knowledge, comparable investigations for indicators of metabolic health have not been published so far. This is despite a considerable overlap in the causative, contraction induced cellular mechanisms and signal transduction pathways that underlay acute and chronic changes in insulin sensitivity [9]. This overlap has to be considered as favorable for an association of effect magnitude. 

The present study aims to investigate the potential of acute reactions to a single bout of exercise to predict chronic training induced improvements in metabolic health. The main hypotheses are associations between acute and chronic exercise induced changes in indicators of insulin sensitivity and in blood lipid parameters, respectively. 

## Material and Methods

### General Design

The present pilot study was designed as an uncontrolled training trial (trial registry: www.clinicaltrials.gov; ID: NCT00934206). It was carried out in accordance with the declaration of Helsinki and approved by the local ethics committee (Ärztekammer des Saarlandes, Saarbrücken, Germany; approval number: 97/09). All participants provided written informed consent prior to participation. The study was carried out in a university department. The total duration (begin of recruitment to last-subject-out) was 9 months. 

To allow for the determination of acute effects the standard design for a training study (two examinations prior to and after a training period) was combined with follow-up measurements during the post-exercise period. These examinations were scheduled 1 and 24 hours after cessation of the initial test. The final test was conducted at day 3 after the last exercise bout of the training period. This time lag has been chosen because it is clearly longer than the acute, exercise induced metabolic alterations [10] while still avoiding detraining effects [11]. 

The protocol for this trial and supporting TREND checklist are available as supporting information; see Checklist S1 and Protocol S1.

### Subjects

Participants were recruited via local media advertisement. Inclusion criteria were selected to represent the typical participant in preventive training: Age 30-60 years, Body mass index 25-35 kg*m^-2^, untrained status (sedentary profession and physical inactivity (

< 1 hour*week^-1^ regular activity; VO_2max_ < 45 ml·min^-1^·kg^-1^, no history of competitive training), non-smoker for at least 1 year, no history of relevant diseases or disorders that would interfere with the intended training (fasting blood glucose concentration < 6.1 mmol*l^-1^, resting blood pressure < 150/95 mmHg). 14 subjects met the above criteria and were included in the study

### Main outcome measures

Fasting indicators of insulin sensitivity and standard blood lipid parameters have been selected as main outcome measures. Additionally, non-esterified fatty acid (NEFA) concentration has been determined because of its potential key role for metabolic training effects. Acute effects of exercise (1 hour after cessation of exercise: AEE_1_; 24 hours after cessation of exercise: AEE_24_) and chronic training effects (CTE) have been calculated by subtracting the initial resting value from the respective measurement. However, this common approach may potentially lead to an overestimation of association between AEE and CTE due to regression to the mean and mathematical coupling [12]. Therefore, main positive findings have been verified by calculating AEE*_1_ and CTE* using the mean of resting values from initial and final test, respectively instead of the initial resting value. 

Fasting blood samples were obtained from the antecubital vein in the supine position. After centrifugation samples were immediately frozen and stored at -20°C. All samples were analyzed in one approach immediately after completion of the training study (UniCell DxC 600 Synchron, Beckmann Coulter GmbH, Germany). From the concentrations of insulin and glucose HOMA-IR has been calculated [13]. A reasonable linear correlation between HOMA-IR and insulin sensitivity as measured by hyperinsulinemic euglycemic glucose clamp tests has been reported in different populations [14].

### Exercise testing and post-exercise period

Exhaustive exercise tests were carried out on a motor-driven treadmill (Woodway ELG 70, Woodway GmbH, Germany) as previously published [7]. In Brief an initial stepwise phase allowing for the determination of lactate concentrations was combined with a final ramp-shaped phase to enable the valid determination of VO_2max_ [15]. Gas exchange measurements (MetaMax2, Cortex, Leipzig, Germany) and 12 lead ECG were carried out throughout the exercise test. Treadmill incline was held constant at 0.5 degrees. The individual protocol from the initial test (initial velocity and number of steps) was held constant for the final test. From gas exchange measurements maximum oxygen uptake (VO_2max_) and maximum respiratory exchange ratio (RER_max_) were determined. 

The post-exercise period has been standardized. Immediately after cessation of exercise the participants sat down and rested in the sitting position for 10 min. Thereafter participants changed to dry clothing but no shower was allowed. The remaining time until the measurements 60 min after cessation of exercise was spent in the recumbent position in a quiet room. Water was allowed ad libitum but no other beverage or nutrient intake.

### Training intervention

Training was carried out over a period of 4 weeks with 4 training sessions per week of 45 min each. Training consisted of walking / running at 60 % of heart rate reserve or the heart rate equivalent to a lactate concentration of basal lactate + 1.5 mmol*l^-1^ (higher value of both prescriptions). Similar protocols have proven to combine effectiveness with compliance in former studies [16]. Participants were equipped with a heart rate monitor programmed with their individual heart rate limits ± 5 min^-1^. Each session was stored in the heart rate monitor for later analysis and control of compliance. Participants were required to attend at least one supervised training session per week. At this occasion training and heart rate logs were checked. 

### Nutritional controls

Care has been taken to limit confounding influences from nutritional variation. All examinations were conducted with participants being in the fasting state for at least 12 hours. The initial and final tests of each individual were scheduled at the same hour between 8 and 11 a.m.. Subjects were required to document their nutrition the day before the initial test and to eat accordingly the day before subsequent visits to the laboratory. From 1 hour prior to training sessions until 1 hour after cessation of training only the intake of water was allowed to control for the possible influence of glucose availability on training efficacy [17]. Apart from the above requirements participants were asked to maintain their previous nutritional habits. 

### Statistics

Normal distribution was checked by Shapiro-Wilk’s test. Acute and chronic changes in outcome measures were tested for significance using one-factorial repeated measures analysis of variance (ANOVA). For post-hoc comparisons the Scheffé test was used where appropriate. Courses of blood lactate concentrations during the initial and final exercise tests were compared using two-factorial ANOVA (factor 1: test 2: step). Associations between AEE and CTE were analyzed by Pearson product-moment correlation. The significance level for the α-error was set at p<0,05. Data are presented as means ± standard deviation.

Multiple regression analysis was not performed due to the limited sample size [18].

## Results

### Drop-outs and compliance

From 14 participants included in the study 2 were unable to complete the training period due to health-related reasons that were independent from the study intervention . From the remaining 12 (♀:n=8; ♂:n=4; age 49 ± 7 years; BMI 29 ± 4; body fat 23 ± 4 %; VO_2max_ 34 ± 7 ml·min^-1^·kg^-1^) 1 refused gas exchange measurements during the final test due to anxiety under the mask. 2 participants did not spend maximum effort in the final test and parameters of exhaustion remained clearly below the corresponding values from the initial test (maximum heart rate 160 vs. 145 min^-1^ and 175 vs. 153 min^-1^, respectively). However, cessation of exercise occurred late enough during the ramp shaped phase of the test that these individuals were eligible for the analysis of submaximal ergometrical measures. 

Adherence to training prescriptions was excellent (100 %). The 12 participants who were included in the analysis completed their training period without relevant deviations from the study protocol as documented by analyzing the files from the heart rate monitors and training logs. In particular, they completed 16 training sessions with a mean heart rate within the prescribed range. 

### Physical performance

**Figure 1:**
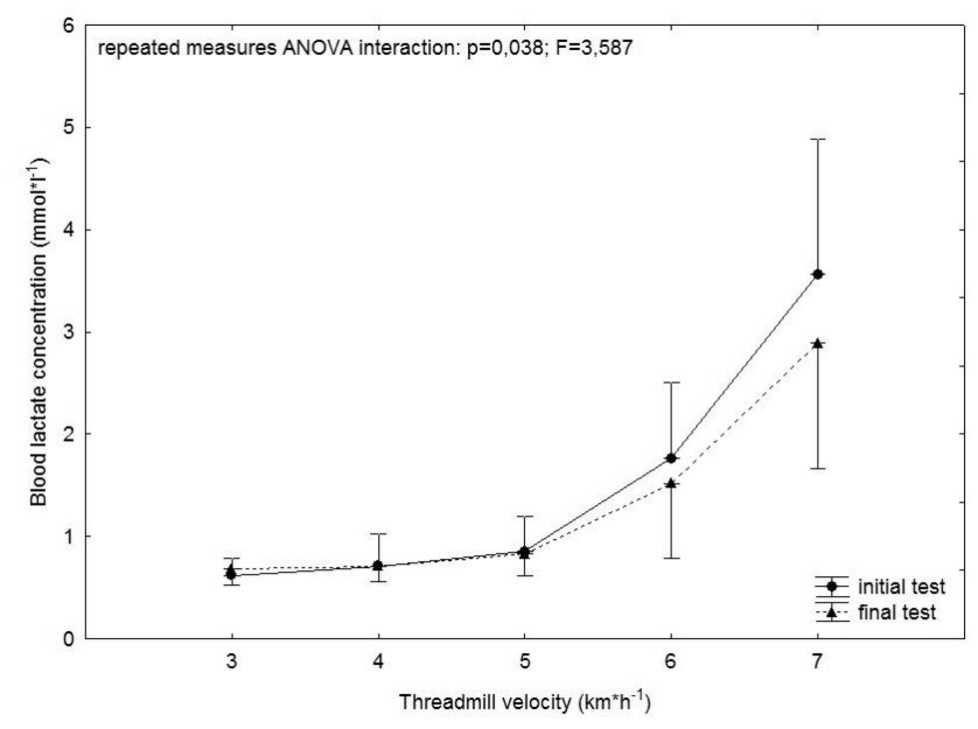
Courses of blood lactate concentrations during exercise testing. Means ± standard deviations.

Submaximal parameters of physical performance improved significantly (n=12; anaerobic threshold 15 ± 3 vs. 17 ± 3 ml·min^-1^·kg^-1^, p=0,014; courses of blood lactate concentration during incremental exercise testing: interaction test*step p=0.038; F=3.587; Figure 1). In contrast, VO_2max_ did not change over the training period (n=9; initial test: 34 ± 7 ml*kg^-1^*min^-1^; final test: 34 ± 7 ml*kg^-1^*min^-1^). Criteria of exhaustion were similar for both tests. Resting heart rate and body mass did not change significantly.

### Magnitude of acute and chronic effects

Significant acute changes from pre-exercise to 1 hour after exercise (AEE_1_) could be demonstrated for all measures of glucose homeostasis (glucose: -0.5 ± 0.4 mmol*l^-1^ p=0.006; insulin: -3.2 ± 2.4 mU*l^-1^ p<0,001; HOMA-IR: -0.8 ± 0.6 p<0.001) and non-esterified fatty acid concentration (-0.23 ± 0.26 mg*dl^-1^ p<0,001). Chronic training effects (CTE) were significant for insulin concentration (-1.7 ± 1.9 mU*l^-1^ p=0.015) and several standard blood lipid parameters (total cholesterol: -15 ± 23 mg*dl^-^1 p=0.040; low density lipoproteins (LDL): -17 ± 14 mg*dl^-1^ p=0.001; triglycerides: -23 ± 40 mg*dl^-1^ p=0.026) while a mean numerical decrease in HOMA-IR of just under half the AEE_1_ failed to reach statistical significance (-0.3 ± 0.6 n.s.). An overview over original readings for the main outcome parameters is given in table 1 (glucose homeostasis) and table 2 (blood lipids and non-esterified fatty acids). 

**Table 1:** Indicators of glucose homeostasis and insulin sensitivity.

			p (vs. initial value)
**Glucose** [mmol*l^-1^]			
Initial resting value		5.5 ± 0.9	n.a.
1 hour after initial test		5.1 ± 0.8	**0.006**
24 hours after initial test		5.8 ± 0.8	0.147
Final resting value		5.7 ± 1.0	0.337
**Insulin** [mU* l^-1^]			
Initial resting value	8.2 ± 3.8		n.a.
1 hour after initial test	5.1 ± 2.1		**<0.001**
24 hours after initial test	8.1 ± 3.6		0.980
Final resting value	6.5 ± 2.8		**0.015**
**HOMA-IR**			
Initial resting value	2.0 ± 1.0		n.a.
1 hour after initial test	1.1 ± 0.5		**<0.001**
24 hours after initial test	2.0 ± 0.9		0.987
Final resting value	1.6 ± 0.7		0.137

HOMA-IR = [(Insulin [mU/l]) * (log glucose [mmol/l]) / 22.5];

Values are means ± SD

Significance testing: one-factorial repeated measures analysis of variance (factor: timepoint of blood collection; post-hoc: Scheffé test).

**Table 2:** Blood lipids and non-esterified fatty acids.

		p (vs. initial value)
**Cholesterol** (mg*dl^-1^ )		
Initial resting value	239 ± 44	n.a.
1 hour after initial test	228 ± 44	0.300
24 hours after initial test	230 ± 45	0.421
Final resting value	223 ± 52	**0.025**
**HDL**2 (mg* dl^-1^ )		
Initial resting value	54 ± 18	n.a.
1 hour after initial test	52 ± 16	0.434
24 hours after initial test	52 ± 15	0.458
Final resting value	55 ± 22	0.995
**LDL** (mg* dl^-1^ )		
Initial resting value	160 ± 36	n.a.
1 hour after initial test	154 ± 35	0.341
24 hours after initial test	158 ± 40	0.850
Final resting value	143 ± 40	**<0.001**
**Triglyceride** (mg* dl^-1^ )		
Initial resting value	131 ± 69	n.a.
1 hour after initial test	114 ± 66	0.189
24 hours after initial test	110 ± 55	0.061
Final resting value	103 ± 53	**0.006**
**NEFA** (mg* dl^-1^ )		
Initial resting value	0.74 ± 0,23	n.a.
1 hour after initial test	0.48 ± 0,18	**<0.001**
24 hours after initial test	0.35 ± 0,17	**<0.001**
Final resting value	0.41 ± 0,12	**<0.001**

HDL: high density lipoprotein cholesterol; LDL: low density lipoprotein cholesterol; NEFA: non-esterified fatty acids; Values are means ± SD

Significance testing: one-factorial repeated measures analysis of variance (factor: timepoint of blood collection; post-hoc: Scheffé test).

### Associations between acute and chronic effects

**Figure 2:**
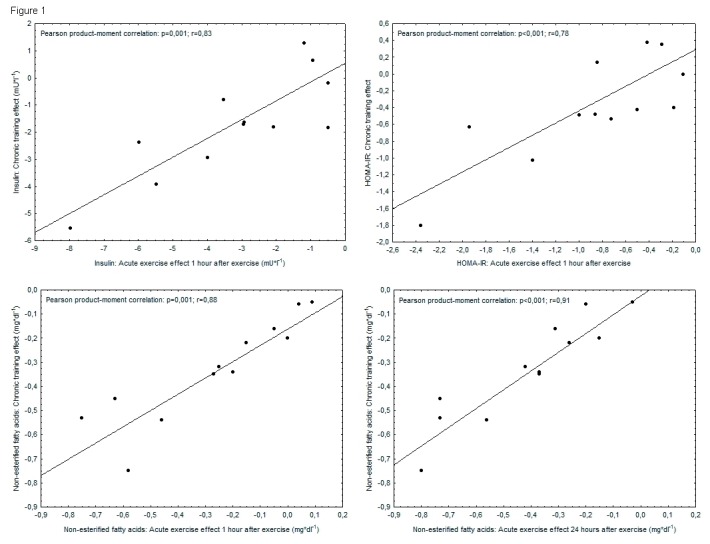
Associations between acute and chronic changes in outcome measures. Upper left: Insulin concentration; Independent variable: acute changes 1 hour after exercise. Upper right: HOMA-IR; Independent variable: acute changes 1 hour after exercise. Lower left: Non-esterified fatty acid concentration; Independent variable: acute changes 1 hour after exercise. Lower right: Non-esterified fatty acids; Independent variable: acute changes 24 hours after . exercise.

**Figure 3:**
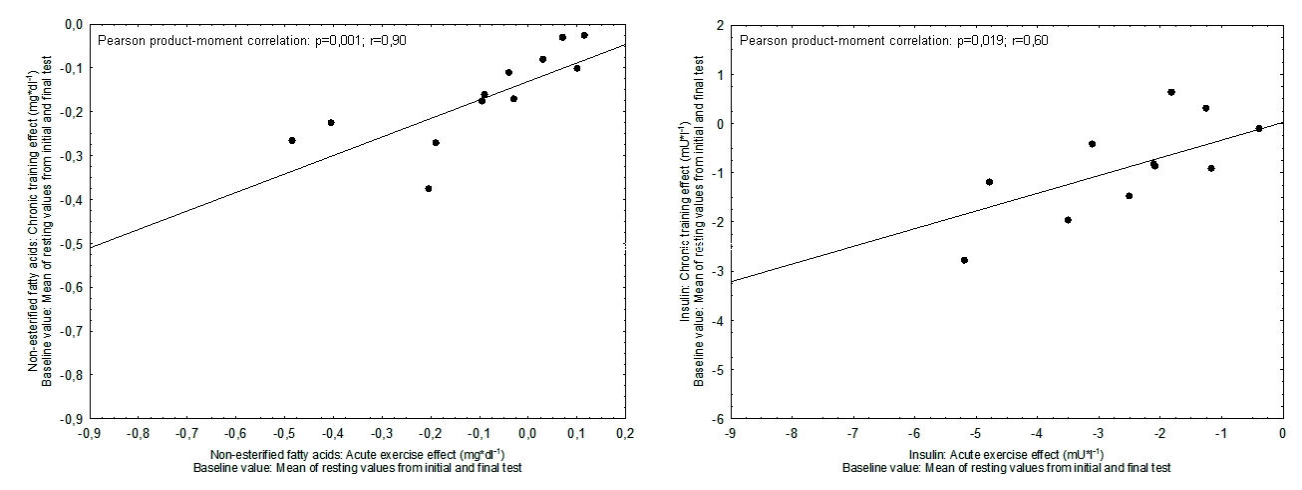
Associations between AEE*_1_ and CTE*. AEE*_1_: Difference between the measurement 1 hour after cessation of exercise in the initial test and the mean of resting values from initial and final tests, respectively (post exercise measurement minus mean of resting values). CTE*: Difference between the resting value in the final test and the mean of resting values from initial and final tests, respectively (resting value from final test measurement minus mean of resting values). Left: Non-esterified fatty acid concentration. Right: Insulin concentration.

For non-esterified fatty acids close linear associations could be demonstrated between AEE_1_ as well as AEE_24_, respectively and the chronic training effect (AEE_1_: p=0.001; r=0.88; AEE_24_: p<0,001; r=0.91). For insulin concentration and HOMA-IR AEE_1_ but not AEE_24_ were significantly associated with the respective training effect (insulin concentration: p=0.001; r=0.83; HOMA-IR: p<0.001; r=0.78). Scatter plots for these associations are shown in Figure 2. With respect to glucose concentration a borderline association between AEE_24_ and CTE could be observed (p=0.046; r=0.58). Associations for AEE*_1_ and CTE* (calculated using the mean of resting values from initial and final tests, respectively as baseline estimate [12], see above) did differ only slightly (Figure 3). 

Neither cholesterol and its subfractions nor triglycerides showed associations between the respective acute and chronic changes.

## Discussion

The main hypothesis of the present study was an association between acute, exercise-induced changes (AEE) in metabolic parameters indicative of cardiovascular risk and their chronic changes after an endurance training program (CTE). This hypothesis could be verified for the concentrations of insulin and glucose, the index of insulin sensitivity HOMA-IR derived from these values and non-esterified fatty acids (NEFA) concentration. These results parallel recently described associations between AEE and CTE for resting blood pressure [7,8]. By contrast, for standard blood lipid parameters no association between AEE and CTE could be verified. 

With respect to the aforementioned studies on AEE and CTE in blood pressure [7,8] it is striking, that acute changes after exhaustive exercise testing [7] as well as after a submaximal, training-type bout of exercise [8] are associated with chronic training effects. Although the second scenario has not been investigated for indicators of metabolic health so far, this is in favor of a robust association between acute and chronic effects of exercise. 

The common and intuitive approach for assessing changes in a continuous outcome is to calculate the difference between the pre- and post-intervention measurements (post-intervention minus pre-intervention). This approach has also been applied in the present study as well as in recent work on AEE and CTE in blood pressure [7,8]. However, this may potentially lead to an overestimation of correlation coefficients due to regression to the mean and mathematical coupling. This consideration has been published for associations between changes and the respective baseline value [12] but is also relevant for associations between acute and chronic changes because the initial resting value has been used in the calculation of AEE as well as CTE. Therefore, main positive findings have been controlled by using the mean of resting values from initial and final tests for the calculation of acute and chronic changes as suggested in [12] (Figure 3). Only minor numerical differences in correlation coefficients were observed as compared to the standard calculation.

Beyond statistics, a physiological coherence as basis of the observed associations would increase the plausibility of acute changes in insulin sensitivity as potential predictors of chronic training effects. While the direct mechanisms of increased glucose uptake into skeletal muscle differ considerably between the two conditions, the causative contraction induced cellular changes and key elements of signal transduction are similar [9,19]. In particular, AMP-activated protein kinase seems to be a causal link to both processes [20–22]. A second parallel may be the negative influence of NEFA concentration on insulin sensitivity [23]. Our data demonstrate a close linear association between NEFA AEE and CTE. Moreover, CTE for NEFA and HOMA-IR, respectively were significantly correlated (p=0.034; r=0.61). 

### Limitations

As a consequence of the limited resources for a pilot study, the number of participants and the duration of the training period were rather low. Due to the resulting numerical relationship between participants and putative predictors a valid multiple regression analysis was not possible and the analysis had to be confined to associations between acute and chronic effects. However, several reasons favor the validity of our results: 1. The main outcome measures have been objectively determined and show acute and chronic effects within the expected range for our specific intervention [24,25]. 2. The timing of examinations allows for a clear differentiation between acute and chronic effects [10,11]. 3. The observed associations do not seem to rely on outliers (Figure 2). 

Another limitation concerns the potential over-reporting of significant findings due to the multiple outcomes in this study. However, even after correcting p-values for the resulting accumulation of α-error by Bonferroni correction [26] the associations of acute and chronic changes for insulin, HOMA-IR and NEFA remain significant. 

Therefore, preliminary acceptance of the main hypothesis seems justified. Randomized controlled follow-up studies with higher n, longer intervention periods and a multivariate regression analysis are needed to confirm our results and evaluate predictive accuracy of acute changes. Also, transferability to different populations and training modes has to be verified.

## Conclusions

Acute, exercise induced changes in insulin sensitivity are promising candidates for the prediction of the respective chronic training effect. This parallels recent findings for resting blood pressure. If these findings can be substantiated and detailed in further studies, AEE may serve as practicable indicators of the individual’s responsiveness to physical exercise. This might offer a significant contribution towards the individual optimization of training prescriptions for preventive training. Moreover, AEE might be used as valuable stratification criterion in training studies thereby increasing internal validity.

## Supporting Information

Checklist S1 **TREND Checklist.**
(PDF)

Protocol S1 **Trial Protocol.**
(DOC)
