# Supplementary material for: Associations between Acute and Chronic Effects of Exercise on Indicators of Metabolic Health: A Pilot Training Trial
Source: PLoS One. 2013 Nov 21;8(11):e81181. doi: 10.1371/journal.pone.0081181 (PMC3836744; doi:10.1371/journal.pone.0081181)
Supplement: Protocol S1 — Trial Protocol. (DOC) [file pone.0081181.s002.doc]

### Fachbereich Klinische Medizin der Universität des Saarlandes

Institut für Sport- und Präventivmedizin

Leiter: Univ.-Prof. Dr. med. T. Meyer

**
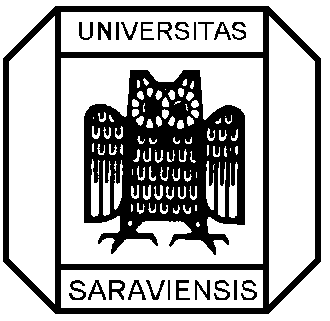
**

|  |  |  |  |
| --- | --- | --- | --- |

## Pilot study: Predictors of training effects in preventive training

The basis for the recommendation of physical activity for disease prevention is constituted by the dose-dependent association of physical activity and physical fitness with lower morbidity and mortality as well as with improvements in cardiovascular risk factors. However, the magnitude of training effects (on fitness as well as on health indicators) varies widely, even within homogeneous groups. This includes so called „low-„ and even „non-responders“. So far, the ex ante estimation of training effects for a specific individual is not possible. Though, in search for predictors it is noteworthy that in in particular blood pressure and insulin sensitivity show acute changes after a single bout of exercise that are similar to the more sustained changes after adherence to a training program. Moreover, the physiological mechanisms of acute and chronic effects (as far as they are known) show considerable overlap. Therefore it seems plausible to suppose an association of acute and chronic effects of exercise.

**Hypotheses:**

Blood pressure: The short term reduction in blood pressure after a single bout of physical exercise („post exercise hypotension“) is associated with the reduction in (resting and exercise) blood pressure after an exercise training program.

Insulin sensitivity and glucose homeostasis: The short term improvement in insulin sensitivity and glucose homeostasis is associated with the respective improvements after an exercise training program.

**Expected effect sizes**:

Expected (Cohen) effect sizes for main outcome measures (acute and chronic): 1

Expected magnitude of acute and chronic changes:

Blood pressure:

Acute: Systolic and diastolic -5 mmHg

Chronic: Systolic -3, diastolic -2 mmHg

Glucose homeostasis (Fasting):

Acute: HOMA-IR -0,8

Glucose concentration: unverändert

Insulin concentration: up to -30 % (depend. on exercise intensity and duration)

Trainingsbedingt: HOMA-IR -1,0

Glucose concentration - 3 mg/dl;

Insulin concentration: up to – 35 % (depend. on exercise intensity and duration)

**Subjects:**

15 healthy, slightly overweight, sedentary volunteers (fasting glucose concentration < 110 mg/dl, resting blood pressure < 140 / 90 mmHg, BMI 25-30 kg/m^2^, VO_2max_ <45 ml·min^-1^·kg^-1^; max. 1x planned physical activity per week, no history of competetive training, sedentary profession), age range 30 und 60 Jahren (target number of complete datasets: 10),

**Study devolution:**

1. Initial test Medical history taking and examination

Antropometry, resting-ECG, blood pressure (4 measurements)

Venous blood sampling

Exhaustive exercise test with gas exchange measurement (see below)

Standardized post exercise periode (10 min sitting, 50 min recumbent)

30’, 45’ und 60’p blood pressure (4x) and heart rate

60’p venous blood sampling

1.b. Late post-exercise 24 hours after the initial test: venous blood sampling and

resting blood pressure

2. Training phase 4 X per week 45 min walking/running for 4 weeks

Intensity: 60 % heart rate reserve bzw. + 1,5 mmol/l (± 5 / min)

No intake of carbohydrate during from 1 h pre-exercise to 60’ p

Control: Heart rate monitor with memory function, training diary, weekly supervised training.

3. Final test 3 days after last exercise bout; same time of day as for initial test, Short medical history taking and examination

Antropometry, resting-ECG, blood pressure (4 measurements)

Venous blood sampling

Echocardiography

Exhaustive exercise test with gas exchange measurement (treadmill)

**Exercise testing protocol**:

Combined step- and rampshaped test on a motor driven treadmill with continuous gas exchange measurement (incline 0,5%; start velocity 4,0, 5,0 oder 6,0 km/h; step increment 1 km/h; step duration 3 min, after presumed transgression of basal lactate ca. + 1,0 mmol/l (ca. 4 Stufen) one more step, then ramp-shaped increase in velocity with increment: 0,8 km/h/min until exhaustion).

**Standardisation measures:**

All test are conducted in the fasting state:

Day before tests Nutrition protocol before initial test

Similar nutrition (incl. Protocol) before following tests

Abundant intake of water

No physical activity

Testing days : Asking for adherence to requirements

Climatized laboratory (23 °C), No insolation

10 min recumbent resting period before measurements

Post exercise 10 min sitting, change to dry clothing (no shower), recumbent waiting period in quiet room until `60 p, water but no other beverage or food

**Statistics:** If main outcome measures are normally distributed differences will be tested by ANOVA and associations by Pearson product-moment correlation.

**Durchführung:**

Responsible: A. Krieg

Doctoral student: T. Grütters

Dr. Anne Krieg Prof. Dr. T. Meyer

**Laborchemie**

|  |  | BB | Entzündung | Fette | Blutdruck | Glucoseh. |
| --- | --- | --- | --- | --- | --- | --- |
| 1.1. | Eingangstest, Ruhe | x | x | x | x | x |
| 1.2. | Eingangstest  60’ p | X | x | x | x | x |
| 1.3. | Eingangstest  Folgemorgen | x | x | x | x | x |
|  | | | | | | |
| 2.1. | Modelltraining  Ruhe | x | x | x | x | x |
| 2.2. | Modelltraining  60’ p | x | x | x | x | x |
| 2.3. | Modelltraining  Folgemorgen | x | x | x | x | x |
|  | | | | | | |
| 4.1. | Abschluss  1. Tag p | x | x | x | x | x |
| 4.2. | Abschluss  2. Tag p | x | x | x | x | x |
| 4.3. | Abschluss  3. Tag p | x | x | x | x | x |

BB: **Blutbild**

Entzündung: **hs-CRP**, TNFα, **IL6**

Fette: **Cholesterin, HDL, LDL, TG**, **NEFA**

Blutdruck: Renin, Aldosteron, Noradrenalin

Glucoseh.: **Glucose, Insulin**, Glucagon
